# Accelerating the Orthodontic Treatment Using Periodontally Accelerated Osteogenic Orthodontics (PAOO): A Periodontic-Orthodontic Interrelationship

**DOI:** 10.7759/cureus.62216

**Published:** 2024-06-12

**Authors:** Ranjith Mari, Rudhra K, Mohan Valiathan, Angelin Fiona J, Sajid Hussain, Anitha Balaji

**Affiliations:** 1 Periodontics, Sree Balaji Dental College and Hospital, Chennai, IND

**Keywords:** root resorption, corticotomy, paoo, osteogenesis, tooth movement, periodontium

## Abstract

Periodontally accelerated osteogenic orthodontics (PAOO) is a periodontal-orthodontic interrelationship procedure that helps in accelerating orthodontic treatment by periodontal procedure reinforcement. This depends on the principle of the regional acceleratory phenomenon which involves inducing changes in the biology of periodontal tissues to fasten the orthodontic tooth movement by creating a surge in the osteopenic environment for tooth movement followed by bone deposition and mineralisation to stabilise the tooth in newly moved position. This PAOO involves the intentional creation of surgical corticotomy cuts followed by a grafting procedure to maintain bone resorption and thickness. Numerous modifications have been incorporated to reduce surgical complications and to improve treatment results by minimally invasive techniques. Hence, this case report incorporated piezosurgery-assisted corticotomy cuts involving the buccal side along with particulate bone grafting to fasten the orthodontic tooth movement, reducing the overall treatment time, root resorption and stabilising the orthodontic treatment results.

## Introduction

Orthodontic tooth movement is intricately linked to periodontal health, making it challenging to maintain periodontal integrity during long-term tooth adjustments due to the involvement of periodontal tissues. To address this issue, Dr. William Wilcko (an orthodontist) and Dr. Thomas Wilcko (a periodontist) developed periodontally accelerated osteogenic orthodontics (PAOO) effective in treating difficult orthodontic tooth movement. This interdisciplinary clinical technique combines selective alveolar corticotomy with particulate bone grafting and subsequent application of orthodontic forces [1]. The primary goal of PAOO is to expedite tooth movement while improving various orthodontic treatments. By merging surgical and orthodontic procedures, PAOO leverages the regional acceleratory phenomenon (RAP) which is a biological mechanism involving basic multicellular units (BMUs) activating the remodelling space to significantly speed up the tooth movement [2]. This phenomenon facilitates faster tooth movement by stimulating RAP which refers to the increased metabolic activity and accelerated bone remodelling in response to surgical trauma. The advantages of this technique include shorter treatment time which fastens the procedure by two to three times, increased alveolar bone width, post-treatment stability, and decreased alveolar bone dehiscence [3]. PAOO is applied in cases of canine retraction, space closure, bimaxillary protrusion, and aesthetic enhancement by gummy smile correction or other esthetic concerns [4].

Periodontists play a crucial part in the PAOO by assisting the orthodontist expedite treatment to get stable results and comprehending the technique's biology to meet patient outcomes [5]. PAOO includes the use of laser technology, modified corticotomy technique, gingival augmentation approach, piezocision technique, Propel Alveolar micro-osteoperforation (MOP) technique, and traditional surgical approach. The objective of this case study is to achieve the hastened orthodontic movement for the repair of bimaxillary protrusion with the use of surgical corticotomy combined with particulate grafting.

## Case presentation

**Figure 1:**
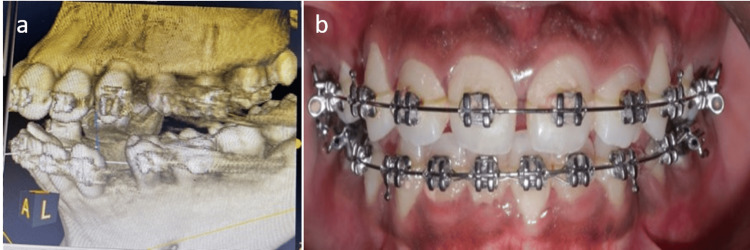
(a) Preoperative cone-beam computed tomography (CBCT) of the patient undergoing orthodontic treatment; (b) clinical preoperative view.

A 24-year-old female patient, undergoing fixed orthodontic treatment for Class I malocclusion with bimaxillary protrusion for one year, presented to our periodontics department. The treatment objectives included correcting the bimaxillary protrusion and improving the dental relationship. To achieve these goals, we planned the extraction of the upper and lower first premolars using maximum anchorage mechanics (Figures 1a-1b).

The PAOO procedure was explained to all patients, and informed consent was obtained for the surgical corticotomies and particulate grafting. For this patient, a corticotomy using a piezoelectric surgery unit was planned to correct the malocclusion, followed by grafting. Blood investigations of complete blood pictures and HbA1c were taken to rule out systemic diseases. Absolute contraindications for this technique are root damage, active periodontal disease, and class III malocclusion.

Surgical procedure

**Figure 2:**
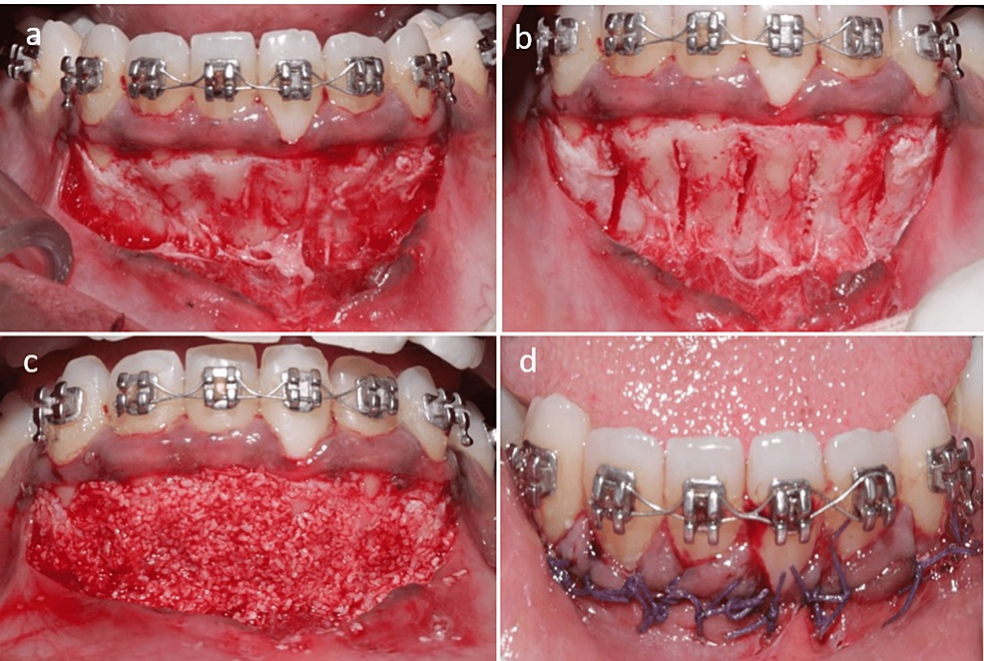
Mandibular periodontally accelerated osteogenic orthodontics (PAOO) procedure (a) Full-thickness flap elevation preserving gingival margin; (b) piezo-assisted corticotomy; (c) particulate bone grafting; (d) suturing done

**Figure 3:**
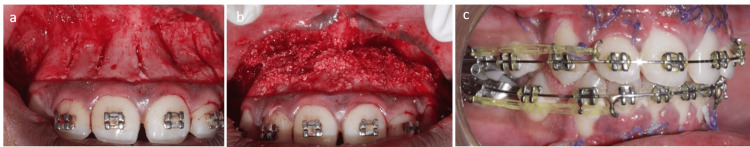
Maxillary periodontally accelerated osteogenic orthodontics (PAOO) procedure (a) Corticotomy; (b) particulate grafting after corticotomy; (c) interrupted Vicryl suture 4-0.

Under local anaesthesia, a full-thickness mucoperiosteal flap was raised while preserving the gingival margin, extending from the lower incisors (13-23, 33-43) beyond the apices (see Figures 2a, 3a).

Corticotomy was performed using a peizosurgical insert (no. OT2) in the areas of 13-23 and 41-43 (refer to Figures 2b, 3b). Vertical cuts were made from 13-23 and 33-43, reaching a depth of about 1.5-2 mm into the cortical bone and extending into the spongiosa. Following hemostasis, an osseograft (particulate bone graft) was placed (as shown in Figures 2c, 3b), and the flap was repositioned and closed using interrupted 4-0 Vicryl sutures (Ethicon, Inc., Bridgewater, USA) (see Figures 2d, 3c).

**Figure 4:**
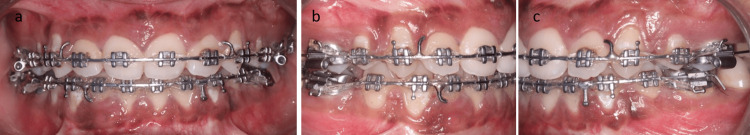
Postoperative view after completion of orthodontic tooth movement (a) Frontal view; (b) right lateral view; (c) left lateral view

Active orthodontic treatment commenced within a week post-surgery. A similar procedure was carried out for the maxillary teeth, with all patients receiving postoperative antibiotics and anti-inflammatory medication for five days. Bimaxillary protrusion correction was achieved within four months (Figures 4a-4c).

## Discussion

Collaboration between orthodontists and periodontists is crucial to determine the necessity of PAOO treatment and to identify the optimal sites for decortication cuts. This collaborative approach ensures an effective perio-ortho treatment protocol tailored to the patient's needs. By discussing and agreeing on the treatment strategy, including the timing and location of decortication cuts, both specialists can align their efforts to achieve the best possible outcomes in terms of periodontal health and orthodontic correction. Previously corticotomy-assisted orthodontics (CAO), MOPs, low-level laser therapy (LLLT), vibration therapy, orthodontic mini-implants (temporary anchorage devices (TADs)), pharmacological agents, piezoelectric surgery, and mechanical vibration devices were used. This joint decision-making process enhances treatment efficiency and promotes long-term stability and aesthetics for the patient's dentition [4].

According to Wilcko et al., to achieve "bone activation," vertical releasing incisions must be made at least one tooth apart. To guarantee appropriate decortication and safeguard the neurovascular complexes leaving the alveolus, flaps must be appropriately reflected past the tooth apices. Creating incisions up to 0.5 mm deep and combining them with selected medullary penetration (corticotomy cuts will engage the cancellous portion to enhance the vascular supply) is known as selective alveolar decortication [5].

Osteotomy, which includes making incisions in the medullary bone surrounding the teeth that are to be moved, is more dangerous than this method and poses a greater risk to the life of the teeth. To the injured bone, an adequate amount of bioabsorbable demineralized bone grafting material (Fix OSS) is applied. Teeth mobility usually starts one to two weeks following surgery [6].

The RAP described by Frost et al. [7] allowed for faster tooth movement in orthodontics which was made possible by corticotomies. Increased osteoblastic activity and bone mineralization assist in stabilising the tooth in its newly shifted position after RAP involvement in alveolar bone induces a spike in osteoclastic activity that lowers bone density and is related to tooth mobility.

Increased activation of BMUs and the creation of woven bone, which will eventually reorganise into lamellar bone, are the cellular and tissue characteristics of RAP. These alterations produce a remodelling space that allows for tooth movement [8]. In order to preserve alveolar bone thickness and decrease the likelihood of root resorption, PAOO is an efficient orthodontic treatment technique that increases the turnover of alveolar spongiosa [9,10]. PAOO, in contrast to corticotomy, simply decorticates, resulting in osteopenia. With the use of orthodontic equipment, this temporary condition causes teeth to move quickly, becoming more malleable and resistant to the force applied by the fixed appliance. The mineralisation within the newly formed bone occurs 20-40 days after this process [11]. Orthopaedic forces are used in buccal and lingual osteotomy cuts as part of a conventional orthodontic treatment protocol that is assisted by a corticotomy. According to recent research, restricting corticotomy to the buccal and labial surfaces may reduce the length of the procedure, the discomfort experienced afterwards, the risk of damaging the lingual architecture, and the likelihood of developing periodontal abnormalities [12].

One of the greatest PAOO procedures is piezocision-assisted corticotomy, which produces predictable periodontal tissue response and good aesthetic results with few postoperative problems [13]. Piezocision and corticotomy-assisted procedures are useful therapeutic approaches for quickening canine retraction [14]. Therefore, to reduce surgical problems and improve surgical predictability, piezosurgery-assisted corticotomy cuts were employed.

The effectiveness of grafting results in increased bone density as proved by various CT-based studies and no objective data was demonstrated on the superiority of graft material [15]. This case also utilised particulate grafting after corticotomy to increase the bone thickness and to stabilise the orthodontics tooth movement results.

## Conclusions

The effective use of PAOO to expedite orthodontic treatment for a patient with bimaxillary protrusion was corrected within four months. Planning and carrying out PAOO requires interdisciplinary cooperation between periodontists and orthodontists in order to ensure accurate decortication and successful treatment outcomes. Utilising the RAP, selective alveolar corticotomy, particulate bone grafting, and piezosurgery allowed for quick tooth movement. In addition to greatly reducing the length of the procedure, the PAOO approach minimised problems and improved alveolar bone width and post-treatment stability. This case shows that PAOO can be an adjunctive modality for treating complicated orthodontic challenges, eventually improving periodontal health and patient happiness, thanks to its creative surgical and orthodontic integration.
